# Selenistasis: Epistatic Effects of Selenium on Cardiovascular Phenotype

**DOI:** 10.3390/nu5020340

**Published:** 2013-01-31

**Authors:** Jacob Joseph, Joseph Loscalzo

**Affiliations:** 1 Department of Medicine, VA Boston Healthcare System, Boston, MA 02132, USA; E-Mail: Jacob.joseph@va.gov; 2 Department of Medicine, Brigham and Women’s Hospital, Boston, MA 02115, USA

**Keywords:** selenium, cardiovascular disease, redox balance, methylation, epigenesis, gene expression, heart failure, fibrosis, methionine, homocysteine

## Abstract

Although selenium metabolism is intricately linked to cardiovascular biology and function, and deficiency of selenium is associated with cardiac pathology, utilization of selenium in the prevention and treatment of cardiovascular disease remains an elusive goal. From a reductionist standpoint, the major function of selenium *in vivo* is antioxidant defense via its incorporation as selenocysteine into enzyme families such as glutathione peroxidases and thioredoxin reductases. In addition, selenium compounds are heterogeneous and have complex metabolic fates resulting in effects that are not entirely dependent on selenoprotein expression. This complex biology of selenium *in vivo* may underlie the fact that beneficial effects of selenium supplementation demonstrated in preclinical studies using models of oxidant stress-induced cardiovascular dysfunction, such as ischemia-reperfusion injury and myocardial infarction, have not been consistently observed in clinical trials. In fact, recent studies have yielded data that suggest that unselective supplementation of selenium may, indeed, be harmful. Interesting biologic actions of selenium are its simultaneous effects on redox balance and methylation status, a combination that may influence gene expression. These combined actions may explain some of the biphasic effects seen with low and high doses of selenium, the potentially harmful effects seen in normal individuals, and the beneficial effects noted in preclinical studies of disease. Given the complexity of selenium biology, systems biology approaches may be necessary to reach the goal of optimization of selenium status to promote health and prevent disease.

## 1. Introduction

Selenium is a unique essential element in that it replaces sulfur in cysteine to form the 21st aminoacid selenocysteine and is, thereby, directly incorporated into proteins, as compared to other metals that act as cofactors or prosthetic groups [1]. This unique biology necessitates a complex metabolic fate for dietary sources of selenium before the selenium moiety becomes biologically active. Several selenoproteins subserve antioxidant functions; however, the full biological repertoire of selenoproteins is still incompletely known. Hence, the biology of selenium is complex and interlinked with other metabolic pathways. The antioxidant effects have prompted investigation into the link of selenium deficiency to pro-oxidant pathologic states such as atherothrombotic cardiovascular disease and heart failure.

Numerous investigations have examined the relation of plasma selenium, a simple and likely inadequate measure of body selenium status, to cardiovascular disease. Although several studies have shown that low selenium status is associated with cardiovascular disease and mortality, other studies have shown a lack of relationship. Furthermore, recent studies of selenium supplementation in the generally selenium-replete population of the United States have demonstrated an increased risk of diabetes mellitus in subjects given selenium in modest supplemental doses. Hence, even though selenium is an attractive nutraceutical by virtue of its significant effects on many biological functions relevant to cardiovascular disease prevention, an incomplete understanding of its biology in health and disease prevents its routine use in humans. In this article, we will review the biological and clinical data on the link between selenium and cardiovascular system, and propose some novel approaches to address this important health problem.

## 2. Clinical Evidence Linking Plasma Selenium to Cardiovascular Disease

### 2.1. Selenium Level and Risk of Cardiovascular Disease

Most of the data demonstrating a link between a low plasma selenium level and cardiovascular disease are derived from observational studies in countries with a low dietary intake of selenium. For example, Salonen and colleagues in a study on subjects in eastern Finland have shown that a plasma selenium level <45 μg/L is associated with an increased risk of coronary artery disease and myocardial infarction [2]; interestingly, fertilizers are supplemented with selenium in Finland to attempt to correct this deficiency in the population. Flores-Mateo and colleagues conducted a meta-analysis of 25 observational studies (11 case-control and 14 cohort studies) that had evaluated the association of blood or toenail selenium with coronary risk [3]. Of the 14 cohort studies, only one was from the United States, while none of the case-control studies were from the United States. In this meta-analysis of predominantly low selenium populations, a 50% increase in selenium concentrations was found to be associated with a 24% decrease in the risk of coronary artery disease. Salvini and colleagues, in their study of United States physicians, did not observe a relationship between low selenium level and risk of myocardial infarction [4]. A recent report from the National Health and Nutrition Examination Survey (NHANES) of 1988–1994 demonstrated a U-shaped relationship between serum selenium level and cardiovascular mortality, with a selenium level of 120 μg/L as the nadir of the relationship [5]. 

Keshan disease is a cardiomyopathy endemic to areas in China where low soil selenium content is prevalent. Studies have shown a relationship of this disease to low dietary intake of selenium and low blood or tissue selenium content, and a reduction in the incidence of cardiomyopathy with selenium supplementation [6]. The seasonal variation observed in the incidence of Keshan disease, however, suggested additional factors may be involved in the genesis of the cardiomyopathy. Studies by Beck and colleagues have shown that selenium deficiency can increase the virulence of a strain of coxsackievirus B3 and convert it from an amyocarditic strain to a pathogenic strain that produces extensive cardiac damage [7,8,9,10]. A recent study has also demonstrated that, on multivariate regression analysis, blood selenium level was not a major correlate of Keshan disease [11]; however, in the same study, activity of the selenoprotein glutathione peroxidase-1 was found to be a major risk factor along with residence in an endemic area and family history of Keshan disease. The current data clearly suggest that Keshan disease is a geochemical disease in which selenium deficiency plays a major role; however, additional factors also likely play a role in the genesis of cardiomyopathy and heart failure.

Deficiency of selenium is associated with cardiomyopathy in conditions such as peripartum cardiomyopathy [12] and cardiomyopathy associated with acquired immune deficiency syndrome [13], conditions associated with nutritional imbalance. A small number of studies have also examined the association of blood selenium levels with heart failure in the general population. Oster and colleagues compared selenium levels in 20 patients with congestive heart failure to those obtained from healthy controls [14]. Of the 20 heart failure patients, 6 had selenium levels comparable to controls, while 14 had low selenium levels (mean 47.8 ± 16.2 μg/L). The selenium level was correlated with left ventricular ejection fraction, a measure of cardiac contractility. A more recent study by de Lorgeril and colleagues examined dietary and blood antioxidants in subjects with chronic heart failure [15]. In this study, dietary intake and blood levels of selenium were found to be lower in heart failure patients; interestingly, the selenium level was correlated with peak oxygen consumption, a major determinant of symptoms and prognosis, but not with left ventricular ejection fraction. Similarly, African Americans with heart failure have been shown to have multiple micronutrient deficits, including that of selenium [16]. The most striking association of selenium deficiency with heart failure is that seen in patients receiving parenteral nutrition. Multiple case reports have shown pathological changes similar to those observed in Keshan disease [17,18], and that selenium supplements can reverse the cardiomyopathic process [19]. 

### 2.2. Selenium Level and Cardiometabolic Health

In the United States, where the population is generally selenium-replete with a mean selenium level of 137.1 ± 19.9 μg/L, as reported by the NHANES [20], the correlation between plasma selenium level and metabolic risk factors for cardiovascular disease appears to be positive. For example, a higher plasma selenium level was positively correlated with higher total, LDL, and HDL cholesterol levels; however, selenium level had a U-shaped relation to the triglyceride level [21]. In the case of glycemic health, similar results were observed with a positive correlation of the plasma selenium level with increased risk of diabetes mellitus and higher glucose and glycosylated hemoglobin levels [20]. In the cross-sectional analysis conducted by NHANES, a higher plasma selenium level was also associated with higher values of systolic and diastolic blood pressure [22]. 

The European population has lower selenium intake and lower selenium levels compared to the United States. Studies conducted in Europe have yielded conflicting data on the relation between the plasma selenium level and cardiometabolic health. In British adults, plasma selenium level was positively correlated with total and non-HDL cholesterol, but not with HDL cholesterol [23]. However, a small pilot study conducted in Britain demonstrated that selenium supplementation had modest benefits on reducing total and non-HDL cholesterol [24]. In a Flemish population, an inverse relationship between serum selenium and blood pressure was observed in men; no relationship was seen in women [25]. In a longitudinal 9-year follow-up study of older French subjects, the baseline selenium level was inversely related to the incidence of dysglycemia in men; again no relationship was observed in women [26]. Conversely, in a study in women residing in Northern Italy, a higher intake of selenium was associated with a higher risk of developing diabetes mellitus [27]. As in the case of cardiovascular mortality, a U-shaped relationship between selenium level and metabolic health has been proposed [28]. 

### 2.3. Inconclusive Benefits of Selenium Supplementation

Selenium supplementation given to a population at risk in areas with endemic selenium deficiency and cardiomyopathy has been shown to reduce the incidence (of Keshan disease) in China [6]. The most direct human correlate of a clearly defined selenium deficient state is that which occurs in patients on total parenteral nutrition, as described above. A recent study conducted by Frustaci and colleagues evaluated the causes of cardiomyopathy in 18 obese subjects who had cardiomyopathy and a history of malabsorption after intestinal bypass surgery [29]. Examination of cardiac biopsies showed no evidence of viral infection; however, cardiac selenium and zinc levels, and glutathione peroxidase activity were markedly reduced compared to idiopathic cardiomyopathy patients and controls. Subjects were randomized to receive additional selenium and zinc supplements or to continue on routine heart failure therapy. Repeat myocardial biopsies after 6 months of treatment showed that myocardial selenium and zinc content and glutathione peroxidase activity normalized in the group given supplements, which was accompanied by recovery of myocyte degeneration and autophagy and improvement in cardiac function. A similar study in heart failure not associated with malabsorption was conducted by Witte and colleagues [30]. Thirty heart failure patients were randomized to receive placebo or a combination of high-dose micronutrients (a combination of 15 minerals and vitamins) including 200 μg/day of selenium. After 9 months of treatment, left ventricular volumes were favorably decreased and left ventricular function significantly increased in the intervention group compared to the placebo group. 

The effects of selenium supplementation in the absence of evident cardiac pathology are less conclusive. A meta-analysis by Flores-Mateo and colleagues pooled the results of 6 randomized trials, of which 3 were in subjects with preexisting coronary heart disease, and 4 utilized selenium in combination with other micronutrients [3]. A non-significant 11% reduction in coronary events was observed in subjects taking selenium supplements. Two recent large cancer prevention trials in the selenium-replete United States population have raised questions about the cardiometabolic benefits of selenium supplements. In the Selenium and Vitamin E Cancer Prevention Trial (SELECT), which examined the effect of L-selenomethionine (200 μg/day) on prostate cancer prevention, a non-significant increase in the incidence of type 2 diabetes mellitus was observed [31]. The Nutritional Prevention of Cancer (NPC) trial examined the effect of high-selenium yeast (200 μg selenium/day) on cancer prevention. In this study a significant increase in the incidence of type 2 diabetes was observed with selenium supplementation [32]. 

### 2.4. Is Selenium an Influence on Cardiovascular Health?

The conflicting data presented above raises the question—does selenium status truly affect cardiovascular health? A major issue with the clinical trials is that the commonest measure of selenium status, the plasma selenium level, is an instantaneous measurement that varies with acute dietary intake, and may not reflect selenium levels over longer times. Tissue selenium, such as toenail selenium levels included in a minority of studies, may integrate selenium values over time and provide a less volatile measure akin to using glycosylated hemoglobin to assess longer-term glycemic status. Recent data suggest that measuring tissue selenium, however, may not reflect “true” selenium status, considering the complex metabolism and biology of selenium. For example, Combs and colleagues, in their study of determinants of selenium status in healthy adults, demonstrated that glutathione peroxidase-3 comprised 20% and selenoprotein P 34% of plasma selenium [33]. The remaining 46% of “nonspecific” selenium contributed the bulk of variability in plasma selenium levels. Another issue is that many genetic and environmental influences are likely to affect selenium disposition and biology. In addition to polymorphisms in selenoprotein genes and genes involved in selenium metabolism, other factors such as viral infection and sulfurated amino acid intake may affect selenium status. In the study by Combs and colleagues, the plasma homocysteine level was a significant correlate of selenoprotein P levels [33]. Studies from the laboratories of Uthus and Davis, as well as a recent study from our laboratory, have demonstrated a close relationship between selenium and the methionine-homocysteine cycle [34,35,36,37]. Another issue with selenium supplementation is that the form of supplementation has varied across studies. Even though the doses of supplements are standardized for the amount of selenium contained therein, there could be significant variability in the *in vivo* biologic effect depending on the type of supplement. For example, the organic form selenomethionine, utilized in the SELECT trial, can be incorporated into proteins in place of methionine competing with its metabolism to selenocysteine and therefore, with selenoprotein synthesis [38]. Inorganic forms of selenium such as selenite at high enough concentrations have been shown to promote oxidant stress by generating reactive oxygen species and by depleting reduced glutathione [39,40]. 

Most of these studies are observational or cross-sectional studies and, hence, do not permit us to determine causality. An interesting factor that is not taken into account in most of the above-mentioned studies is that polymorphisms in selenoprotein genes may affect the disposition of selenium and, thereby, cardiovascular outcomes independent of dietary intake. For example, Alanne and coworkers have demonstrated that polymorphisms in the Selenoprotein S gene have significant effects on cardiovascular morbidity, especially in women [41]. The better conclusion drawn from the conflicting data accumulated over the last four decades is that while optimal selenium status is crucial to cardiovascular health, the complex biology of selenium and selenium compounds must be taken into account to estimate and optimize selenium status in humans. There is an urgent need for research into selenium biology in health and disease, considering the high use of selenium supplements in the selenium-replete Unites States population [42]. 

## 3. Biphasic Effects of Selenium Status

### 3.1. Major Biologic Effects of Selenium

Selenium is unique among essential minerals in that it is incorporated into amino-acid peptide structure in place of sulfur to form the aminoacid selenocysteine. Incorporation of selenocysteine into proteins is accomplished via read-through of the termination codon UGA by unique translational machinery [1]. Selenoproteins have a major role in maintaining redox balance (e.g., glutathione peroxidases and thioredoxin reductases). Selenoprotein expression in tissues demonstrates an hierarchical relation, with the liver and heart affected more than endocrine organs and brain by selenium deficiency [43]. Studies also suggest that there is a hierarchy of expression among selenoproteins themselves, with some selenoproteins being more sensitive to selenium level or changes in translational machinery, termed “stress-related” selenoproteins, and others that are less affected, termed “housekeeping” selenoproteins [44,45,46]. There are over 25 mammalian selenoproteins described thus far; the functions of each of these selenoproteins have not been elucidated. 

#### 3.1.1. Anti-Oxidant Effects

Oxidant stress is implicated in many cardiovascular diseases, including atherosclerosis, myocardial infarction, and heart failure [47,48]. Multiple pre-clinical studies have demonstrated that during myocardial injury produced by ischemia and the combination of ischemia and reperfusion, selenoproteins, predominantly glutathione peroxidase-1, reduce oxidant stress. Selenium deficiency worsens, and selenium supplementation abrogates, myocardial injury [49,50,51,52]. In doxorubicin-induced cardiomyopathy, in which oxidant stress plays a major role, selenium deficiency has been shown to worsen cardiac pathology, with the opposite effect observed with selenium supplementation [53,54,55,56,57]. In some studies, however, a beneficial effect of selenium supplementation was not observed [58,59]. The spontaneously hypertensive rat (SHR) is a model of progressive hypertensive remodeling of the myocardium resulting in heart failure in which oxidant stress is thought to play a major role. A recent study showed that normal and high levels of selenium in the diet reduced heart failure-related mortality in SHRs compared to a selenium-deficient diet [60]. 

#### 3.1.2. Cell Proliferation and Survival

Multiple studies have demonstrated anti-angiogenic effects of selenium, a potential mechanism of cancer prevention. For example, in an orthotopic model of human colon cancer in athymic nude mice, selenium supplementation with methylselenocysteine resulted in significant inhibition of microvessel formation and tumor growth [61]. Other studies have also demonstrated similar anti-angiogenic effects of organic selenium supplements in cancer cells and tissues [62,63]. Similar results have also been observed in non-tumorigenic tissues. A transcriptomic analysis in normal rats showed that angiogenic genes were affected by selenite supplementation [64]. However, in diabetic mice, selenium (provided as selenite) has been shown to promote angiogenesis and, thereby, wound healing [65]. Both pro- and anti-apoptotic effects have been demonstrated with selenium supplementation. Methylselenic acid has been shown to promote apoptosis in vascular endothelial cells, in keeping with the anti-angiogenic effect mentioned above [66], while selenite has been demonstrated to attenuate oxysterol-induced apoptosis in vascular smooth muscle cells [67]. 

#### 3.1.3. Immune and Anti-Infective Mechanisms

Selenium and selenoproteins, via redox balance and calcium flux, are linked to effector functions of immune cells [68], which may be important for cardiovascular biology considering the importance of innate and adaptive immunity in the genesis of cardiovascular disease. As mentioned above, the cardiomyopathy observed in Keshan disease may result from increased viral pathogenicity induced by the selenium-deficient state, as shown by a series of investigations by Beck and colleagues. The amyocarditic strain of coxsackievirus B3 was shown to be non-pathogenic in selenium-replete mice while it induced myocarditis in selenium-deficient mice [8]. Furthermore, virus recovered from selenium-deficient mice induced myocarditis when inoculated into selenium-replete mice, clearly indicating a change in virulence of the organism. This increase in virulence was shown to result from six nucleotide changes in the viral genome [9]. There were no changes in natural killer cell activity or neutralizing antibody levels between selenium-replete and -deficient animals [7]. However, lymphocyte proliferation in response to mitogen and antigen were reduced in selenium-deficient mice. Chagas disease, a tropical cardiomyopathy resulting from infection with the parasite *Trypanosoma cruzi*, is also associated with low selenium levels [69]. Preclinical studies have shown that selenium supplementation improves survival in rodents infected with this parasite [70,71]. Gender differences in the effect of selenium on immune function have been described. For example, Stoedter and colleagues have shown that the fulminant immune response induced by lipopolysaccharide is attenuated by selenium supplementation in male mice, but not female mice [72]. 

#### 3.1.4. Effects on Matrix Metabolism

An *in vivo* study in rats showed that high levels of selenium increased the amount of immature collagen in various organs, including the heart [73]. Conversely, selenite decreased stellate cell number and liver fibrosis and increased the expression of matrix metalloproteinase-9 in a model of liver fibrosis [74]. Selenite was also shown to decrease matrix metalloproteinase-13 expression, increase the expression of tissue inhibitors of metalloproteinase-1 and -2, and prevent a decrease in type II collagen induced by T2 toxin in human chondrocytes [75]. Another *in vitro* study also demonstrated a similar effect of selenite in human fibrosarcoma cells [76]. A recent transcriptomic analysis in rodents showed that dietary selenium increased extracellular matrix-associated gene expression [64]. Conversely, knockout of selenocysteine tRNA in myeloid cells led to increased expression of tissue inhibitor of metalloproteinase-3 as well as the genes for types I and III collagens [77]. As matrix composition of the vascular wall and the myocardium is crucial to cardiovascular function, it is possible that selenium may modulate cardiovascular health via effects on matrix metabolism. In fact, a recent study from our laboratories demonstrates a potent effect of both selenium deficiency and modest supplementation on myocardial matrix levels and function [37].

#### 3.1.5. Epigenetic Mechanisms

In addition to the genome, epigenetic mechanisms, or heritable modifications that change chromatin structure and transcriptional activity independent of any change in the DNA sequence, also influence gene transcription. DNA methylation, histone modifications, and non-coding RNAs are the major epigenetic mechanisms that alter chromatin structure between transcriptionally active euchromatin and transcriptionally inactive heterochromatin [78]. Selenium affects DNA methylation and gene transcription both *in vivo* and *in vitro* [79,80]. Dietary selenium supplementation has been shown to cause global DNA hypomethylation associated with hypermethylation of the promoter of the tumor suppressor gene p53 [81]. Selenium also affects the availability of *S*-adenosyl methionine, the methyl donor for all DNA methylation reactions, as well as the concentration of *S*-adenosyl homocysteine, a potent inhibitor of DNA methyltransferases. These effects on methylation potential are mediated by the interaction of metabolic pathways of selenium with the methionine-homocysteine cycle [35,36,82,83], and are described in more detail below. 

### 3.2. Both Selenium Deficiency and Selenium Supplementation Induce Similar Phenotypes

The conflicting clinical data and varied and complex biology described above suggest that the effects of selenium are unlikely to be linear across clinically relevant dose ranges in healthy conditions. In fact several recent studies have demonstrated a biphasic effect of selenium. For example, in mice transgenic for transforming growth factor α/cMyc and prone to develop liver cancer, both selenium deficiency and supplementation (2.5 ppm dietary selenium) inhibited carcinogenesis [84]. Similarly, Labunskyy and colleagues have demonstrated that in normal mice, modest selenium supplementation induced insulin resistance, while genetic suppression of selenoprotein synthesis also induced a diabetic phenotype [44]. A recent study from our laboratories examined the effect of selenium deficiency and modest selenium supplementation on myocardial structure and function [37]. Our results showed that both selenium deficiency and supplementation promoted myocardial fibrosis and diastolic dysfunction compared to normal dietary selenium. Differential changes in redox state and DNA methylation were observed in selenium deficient and excess states; these changes over 12 weeks resulted in similar adverse phenotypes. Selenium deficiency produced a more adverse phenotype with diastolic and systolic dysfunction, while selenium supplementation produced only diastolic dysfunction. 

## 4. Selenium and Redox-Methylation Balance

**Figure 1:**
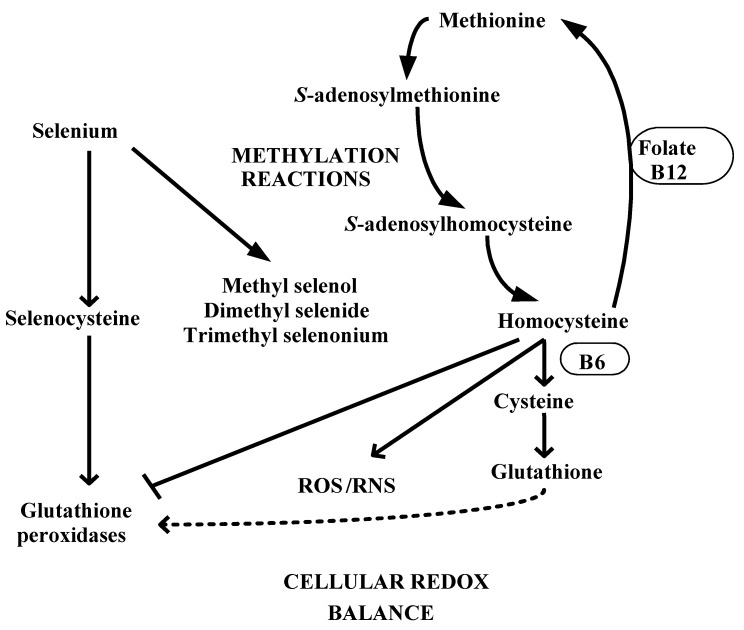
Interaction of selenium and the methionine-homocysteine cycle (reproduced with permission from Metes-Kosik *et al.* [37]). ROS: reactive oxygen species; RNS: reactive nitrogen species.

Multiple lines of evidence suggest a close link between selenium metabolism and the methionine-homocysteine cycle and, thereby, with redox-methylation balance [37]. As shown in Figure 1, the mocysteine cycle starts with methionine, an essential sulfurated aminoacid. Methionine is converted to *S*-adenosyl methionine which provides an active methyl group for all methylation reactions except the remethylation reaction that converts homocysteine to methionine. Loss of the methyl group from *S*-adenosyl methionine leads to production of *S*-adenosyl homocysteine, which is hydrolyzed to homocysteine. Homocysteine is remethylated via a vitamin B_12_- and folate-dependent pathway or by the enzyme betaine homocysteine methyl transferase. An alternate fate of homocysteine is transsulfuration via the vitamin B_6_-dependent enzyme cystathionine beta synthase, which is an irreversible pathway that culminates in the production of cysteine. Cysteine is the precursor of glutathione, the obligate substrate for the crucial cellular antioxidant selenoprotein glutathione peroxidase-1. The relative concentrations of *S*-adenosyl methionine and *S*-adenosyl homocysteine (an inhibitor of DNA methyl transferases) modulate DNA methylation and gene transcription. While homocysteine is considered a pro-oxidant molecule, its conversion to cysteine, the major extracellular antioxidant and glutathione, the major intracellular antioxidant, modulates redox balance and signaling [85].

Figure 1 also demonstrates the close link between selenium metabolism and the methionine-homocysteine cycle. The first link is via glutathione/redox balance. Hill and Burk have shown that selenium deficiency directly stimulates the activity of glutamate cysteine ligase (a rate-limiting enzyme in glutathione synthesis), and thereby, increases glutathione synthesis in liver cells [86,87]. Uthus and colleagues have shown that selenium deficiency decreases total homocysteine levels in rats, although the study could not conclusively prove whether or not this was due to shunting of homocysteine into the transsulfuration pathway towards synthesis of glutathione [34]. The second link is via methylation reactions. As shown in Figure 1, selenium is methylated *in vivo* into methyl selenol, dimethyl selenide, and trimethyl selenonium, and, thereby, can act as a “methyl sink” [88]. In addition, as described above, selenium has complex effects on methylation via both effects on the balance between *S*-adenosyl methionine and *S*-adenosyl homocysteine levels as well as direct effects on DNA methyltransferase activity. In our recent study of the effects of selenium status on myocardial gene regulation, we found a close interaction between selenium status, methionine-homocysteine cycle, and DNA methylation as predicted from the above-mentioned data [37]. While the plasma homocysteine level was reduced by selenium deficiency, selenium supplementation as inorganic selenite initially (3 weeks) reduced the plasma glutathione level with a restoration to normal by 12 weeks. While selenium deficiency did not alter methylation potential or the ratio of *S*-adenosyl methionine to *S*-adenosyl homocysteine, selenium supplementation significantly elevated the level of *S*-adenosyl homocysteine. Selenium supplementation decreased DNA methyltransferase activity at 3 weeks, an effect that was accompanied by a decrease in DNA methylation between 3 and 12 weeks. 

**Figure 2:**
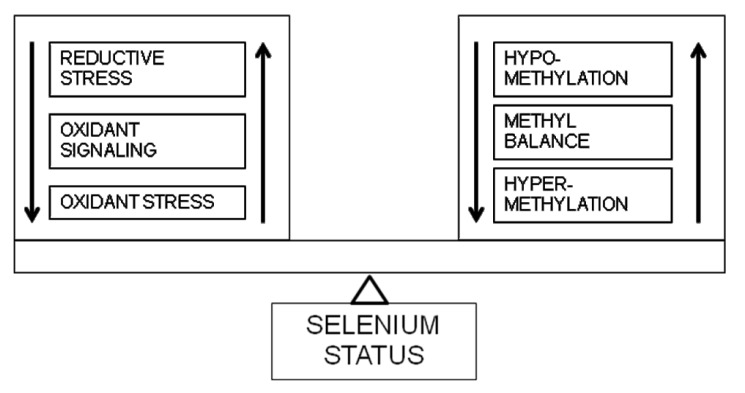
Selenium and redox-methylation balance. Selenium plays a pivotal role in balancing the flux between potential redox and methylation states.

Thus, a potential explanation of the biphasic response to selenium may be its effects on the continuums of redox status and methylation status. As shown in Figure 2, signaling via free radicals is integral to normal cellular function. A preponderance of oxidants or reductants can move the cell into the realms of oxidant stress and reductive stress, respectively. As described in previous sections, there is abundant literature on the role of increased oxidant stress in the genesis of cardiovascular disease. Recent reports suggest that reductive stress is also an important pathogenic mechanism in cardiovascular disease. For example, a cardiac specific mutation in the human αB crystallin gene has been shown to increase cycling of oxidized to reduced glutathione causing reductive stress, resulting in a cardiomyopathic phenotype [89]. Over expression of the selenoprotein glutathione peroxidase-1 in mice leads to hyperglycemia and insulin resistance [90]. In these mice, lower reactive oxygen species levels resulting in over-expression of pancreatic duodenal homeobox 1 and uncoupling protein-2 were thought to underlie the changes in insulin production and hyperinsulinemia [91]. Interestingly, a selenium deficient diet was found to attenuate these metabolic abnormalities in glutathione peroxidase-1 over-expressing mice [92]. Hence, as shown in Figure 2, a sliding scale of redox balance may exist in cells and tissues in which optimal signaling promotes cellular health, while both oxidant and reductive stress may lead to pathology, with selenium status as a potentially crucial determinant of redox state. 

Similar to its effects on redox balance, selenium also influences methylation balance as described above and illustrated in Figure 1. Methylation of CpG dinucleotides in gene promoter regions leads to suppression of gene expression. Hypomethylation has the opposite effect. The effects on gene methylation are variable in the adult state, with generalized DNA hypomethylation found to coexist with hypermethylation of specific genes [81]. As shown in Figure 1, Figure 2, selenium may affect not only the balance between various redox states and different methylation states, but could integrate the redox-methylation balance itself, as shown in our recent study [37]. The effect on redox-methylation balance may explain the variability seen in the effects of selenium in health and disease.

## 5. Selenistasis—A Framework for Selenium in Health and Disease

**Figure 3:**
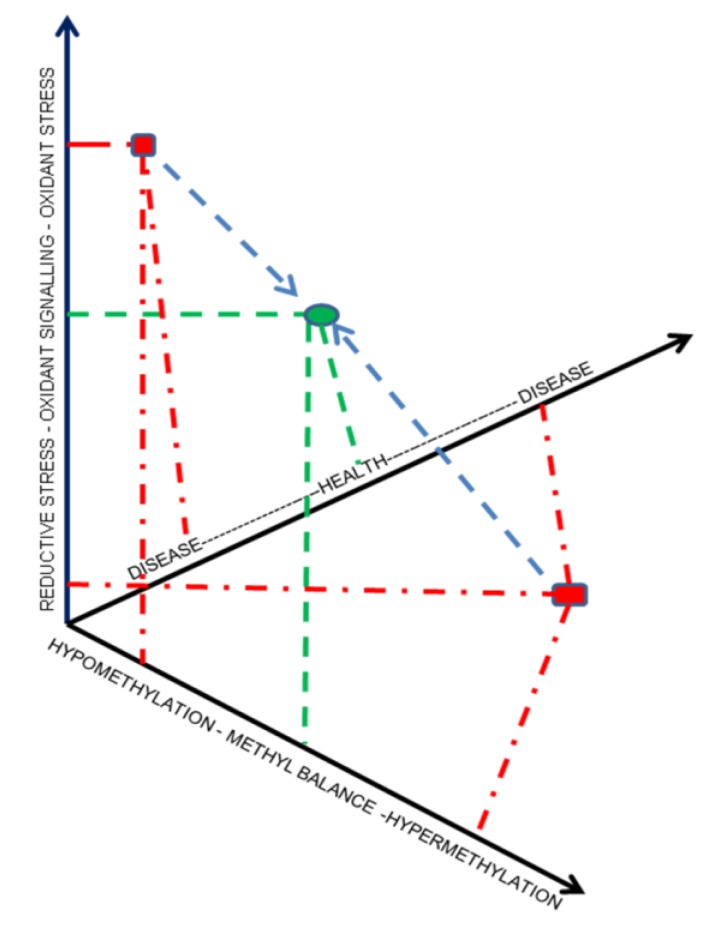
Selenistasis—Selenium integrates redox-methylation balance in health and disease. The X-axis represents methylation status, the Y-axis represents redox state, and the Z-axis represents the continuum from health to disease states. The green oval represents normal oxidant signaling and methylation balance in health. The red square and red rectangle represent two possible combinations of changes in redox and methylation states in disease. Blue arrows delineate the desired effect of interventions in diseased states, *i.e.*, to alter redox and methylation states and, thereby, approximate selenistasis.

Epistasis is a term used to denote an interaction between two genes that results in modification of phenotype [93]. As shown above, selenium acts as an integrator for the reciprocal relation between redox state and methylation status and, thereby, can influence cardiovascular phenotype, a condition similar to epistasis but involving two fundamental cellular biologic processes. We wish to propose the term *selenistasis* to denote this relationship. As shown in Figure 3, selenistasis is defined in relation to the health of the organism. The blue circle shows the combination of normal oxidant signaling and methyl balance in the healthy state, or selenistasis. The actual selenium level is not determinative of selenistasis in this conceptual model, regardless of the measured plasma selenium level, redox and methylation status would determine deviation from “selenistasis”. This construct offers a potential solution to the contentious issue of whether the plasma selenium level truly reflects “selenium status”. 

The red square and rectangle show one of several possible combinations of changes in redox and methylation states in severe disease. Once the underlying redox-methylation state in each disease state is established, changing dietary selenium can be utilized to achieve selenistasis, as shown in Figure 3. This effect may differ with different disease states and in different tissues and cell types. 

## 6. Systems Biology—A Novel and Necessary Approach to Evaluating Selenistasis

The biology of selenium intersects with major cellular processes and is different in healthy *vs.* diseased states. Hence, traditional reductionist methods are unlikely to allow us to understand selenium status and its relationship to health and disease. With rapid advances in -omics technologies in network science, and in systems biology, it is now possible to evaluate the comprehensive biologic effects of varying doses of selenium in normal and disease states. Systems biology allows us to integrate large amounts of data to create a network model that can explain the conflicting results seen in selenium research [94,95]. Efforts are underway to create a collaborative network-based approach to micronutrient biology. The Micronutrient Genomics Project is a community-driven project that captures comprehensive data related to micronutrient genome interactions [96]. As part of this project, a Wikipathway has been created for selenium biology in humans [97]. We propose that systems biology approaches should be used to define a selenistatic network in healthy conditions based on -omics data generated from analysis of normal tissues subjected to varying doses of selenium. Most of the data generated would be from animal models, but human samples, such as toenails, buccal cells, heart biopsies undertaken for clinical reasons, and heart samples obtained during implantation of left ventricular assist devices in patients with advanced heart failure, could also be tissue sources of selenium measurements in humans. This would help establish major hubs of selenistasis and the role of redox-methylation balance in different tissues. Networks should then be established using data obtained from disease states under conditions of normal selenium intake to define deviations from selenistasis under specific conditions in the whole organism (blood measurements) or in specific organs. Further network analysis should be conducted in disease states with varying intake of selenium. Such network medicine-based systems analysis is likely to lead to a better understanding of selenium biology in health and disease and to multiple biomarker-based approaches to measure and optimize selenistasis. 

## 7. Conclusions

Selenium is clearly essential for human health, but how can we optimize selenium status to prevent and treat cardiovascular disease? It is clear that the effects of selenium are complex, cell- and tissue-dependent, and influenced by the disease process itself. It is also clear that this micronutrient is essential for human health. How changes in selenium content of the diet affect biological processes in normal individuals, and how selenium affects pathogenic processes and *vice versa* still needs elucidation. The concepts of redox-methylation balance and selenistasis may give us a framework to understand the complex biology of selenium *in vivo* and develop diagnostic tests to evaluate selenium status and its (patho)biological consequences. The tools of systems biology will be crucial to this endeavor. 

## References

[1] Stadtman T.C. (2005). Selenoproteins—Tracing the role of a trace element in protein function. PLoS Biol..

[2] Salonen J.T., Alfthan G., Huttunen J.K., Pikkarainen J., Puska P. (1982). Association between cardiovascular death and myocardial infarction and serum selenium in a matched-pair longitudinal study. Lancet.

[3] Flores-Mateo G., Navas-Acien A., Pastor-Barriuso R., Guallar E. (2006). Selenium and coronary heart disease: A meta-analysis. Am. J. Clin. Nutr..

[4] Salvini S., Hennekens C.H., Morris J.S., Willett W.C., Stampfer M.J. (1995). Plasma levels of the antioxidant selenium and risk of myocardial infarction among U.S. physicians. Am. J. Cardiol..

[5] Bleys J., Navas-Acien A., Guallar E. (2008). Serum selenium levels and all-cause, cancer, and cardiovascular mortality among us adults. Arch. Intern. Med..

[6] Ge K., Yang G. (1993). The epidemiology of selenium deficiency in the etiological study of endemic diseases in china. Am. J. Clin. Nutr..

[7] Beck M.A., Kolbeck P.C., Shi Q., Rohr L.H., Morris V.C., Levander O.A. (1994). Increased virulence of a human enterovirus (coxsackievirus B3) in selenium-deficient mice. J. Infect. Dis..

[8] Beck M.A., Kolbeck P.C., Rohr L.H., Shi Q., Morris V.C., Levander O.A. (1994). Benign human enterovirus becomes virulent in selenium-deficient mice. J. Med. Virol..

[9] Beck M.A., Shi Q., Morris V.C., Levander O.A. (1995). Rapid genomic evolution of a non-virulent coxsackievirus B3 in selenium-deficient mice results in selection of identical virulent isolates. Nat. Med..

[10] Beck M.A. (1997). Increased virulence of coxsackievirus B3 in mice due to vitamin E or selenium deficiency. J. Nutr..

[11] Lei C., Niu X., Ma X., Wei J. (2011). Is selenium deficiency really the cause of keshan disease?. Environ. Geochem. Health.

[12] Lee S.R., Bar-Noy S., Kwon J., Levine R.L., Stadtman T.C., Rhee S.G. (2000). Mammalian thioredoxin reductase: Oxidation of the *C*-terminal cysteine/selenocysteine active site forms a thioselenide, and replacement of selenium with sulfur markedly reduces catalytic activity. Proc. Natl. Acad. Sci. USA.

[13] Dworkin B.M., Antonecchia P.P., Smith F., Weiss L., Davidian M., Rubin D., Rosenthal W.S. (1989). Reduced cardiac selenium content in the acquired immunodeficiency syndrome. J. Parenter. Enter. Nutr..

[14] Oster O., Prellwitz W., Kasper W., Meinertz T. (1983). Congestive cardiomyopathy and the selenium content of serum. Clin. Chim. Acta.

[15] De Lorgeril M., Salen P., Accominotti M., Cadau M., Steghens J.P., Boucher F., de Leiris J. (2001). Dietary and blood antioxidants in patients with chronic heart failure: Insights into the potential importance of selenium in heart failure. Eur. J. Heart Fail..

[16] Arroyo M., Laguardia S.P., Bhattacharya S.K., Nelson M.D., Johnson P.L., Carbone L.D., Newman K.P., Weber K.T. (2006). Micronutrients in African-Americans with decompensated and compensated heart failure. Transl. Res..

[17] Inoko M., Konishi T., Matsusue S., Kobashi Y. (1998). Midmural fibrosis of left ventricle due to selenium deficiency. Circulation.

[18] Lockitch G., Taylor G.P., Wong L.T., Davidson A.G., Dison P.J., Riddell D., Massing B. (1990). Cardiomyopathy associated with nonendemic selenium deficiency in a caucasian adolescent. Am. J. Clin. Nutr..

[19] Reeves W.C., Marcuard S.P., Willis S.E., Movahed A. (1989). Reversible cardiomyopathy due to selenium deficiency. J. Parenter. Enter. Nutr..

[20] Laclaustra M., Navas-Acien A., Stranges S., Ordovas J.M., Guallar E. (2009). Serum selenium concentrations and diabetes in U.S. Adults: National Health and Nutrition Examination Survey (NHANES) 2003–2004. Environ. Health Perspect..

[21] Laclaustra M., Stranges S., Navas-Acien A., Ordovas J.M., Guallar E. (2010). Serum selenium and serum lipids in US adults: National Health and Nutrition Examination Survey (NHANES) 2003–2004. Atherosclerosis.

[22] Laclaustra M., Navas-Acien A., Stranges S., Ordovas J.M., Guallar E. (2009). Serum selenium concentrations and hypertension in the US population. Circ. Cardiovasc. Qual. Outcomes.

[23] Stranges S., Laclaustra M., Ji C., Cappuccio F.P., Navas-Acien A., Ordovas J.M., Rayman M., Guallar E. (2010). Higher selenium status is associated with adverse blood lipid profile in British adults. J. Nutr..

[24] Rayman M.P., Stranges S., Griffin B.A., Pastor-Barriuso R., Guallar E. (2011). Effect of supplementation with high-selenium yeast on plasma lipids: A randomized trial. Ann. Intern. Med..

[25] Nawrot T.S., Staessen J.A., Roels H.A., Den Hond E., Thijs L., Fagard R.H., Dominiczak A.F., Struijker-Boudier H.A. (2007). Blood pressure and blood selenium: A cross-sectional and longitudinal population study. Eur. Heart J..

[26] Akbaraly T.N., Arnaud J., Rayman M.P., Hininger-Favier I., Roussel A.M., Berr C., Fontbonne A. (2010). Plasma selenium and risk of dysglycemia in an elderly french population: Results from the prospective epidemiology of vascular ageing study. Nutr. Metab..

[27] Stranges S., Sieri S., Vinceti M., Grioni S., Guallar E., Laclaustra M., Muti P., Berrino F., Krogh V. (2010). A prospective study of dietary selenium intake and risk of type 2 diabetes. BMC Public Health.

[28] Rayman M.P. (2012). Selenium and human health. Lancet.

[29] Frustaci A., Sabbioni E., Fortaner S., Farina M., del Torchio R., Tafani M., Morgante E., Ciriolo M.R., Russo M.A., Chimenti C. (2012). Selenium- and zinc-deficient cardiomyopathy in human intestinal malabsorption: Preliminary results of selenium/zinc infusion. Eur. J. Heart Fail..

[30] Witte K.K., Nikitin N.P., Parker A.C., von Haehling S., Volk H.D., Anker S.D., Clark A.L., Cleland J.G. (2005). The effect of micronutrient supplementation on quality-of-life and left ventricular function in elderly patients with chronic heart failure. Eur. Heart J..

[31] Lippman S.M., Klein E.A., Goodman P.J., Lucia M.S., Thompson I.M., Ford L.G., Parnes H.L., Minasian L.M., Gaziano J.M., Hartline J.A. (2009). Effect of selenium and vitamin E on risk of prostate cancer and other cancers: The selenium and vitamin E cancer prevention trial (SELECT). JAMA.

[32] Stranges S., Marshall J.R., Natarajan R., Donahue R.P., Trevisan M., Combs G.F., Cappuccio F.P., Ceriello A., Reid M.E. (2007). Effects of long-term selenium supplementation on the incidence of type 2 diabetes: A randomized trial. Ann. Intern. Med..

[33] Combs G.F., Watts J.C., Jackson M.I., Johnson L.K., Zeng H., Scheett A.J., Uthus E.O., Schomburg L., Hoeg A., Hoefig C.S. (2011). Determinants of selenium status in healthy adults. Nutr. J..

[34] Uthus E.O., Yokoi K., Davis C.D. (2002). Selenium deficiency in Fisher-344 rats decreases plasma and tissue homocysteine concentrations and alters plasma homocysteine and cysteine redox status. J. Nutr..

[35] Uthus E.O., Ross S.A., Davis C.D. (2006). Differential effects of dietary selenium (Se) and folate on methyl metabolism in liver and colon of rats. Biol. Trace Elem. Res..

[36] Davis C.D., Uthus E.O. (2003). Dietary folate and selenium affect dimethylhydrazine-induced aberrant crypt formation, global DNA methylation and one-carbon metabolism in rats. J. Nutr..

[37] Metes-Kosik N., Luptak I., Dibello P.M., Handy D.E., Tang S.S., Zhi H., Qin F., Jacobsen D.W., Loscalzo J., Joseph J. (2012). Both selenium deficiency and modest selenium supplementation lead to myocardial fibrosis in mice via effects on redox-methylation balance. Mol. Nutr. Food Res..

[38] Salbe A.D., Levander O.A. (1990). Comparative toxicity and tissue retention of selenium in methionine-deficient rats fed sodium selenate or L-selenomethionine. J. Nutr..

[39] Xiang N., Zhao R., Zhong W. (2009). Sodium selenite induces apoptosis by generation of superoxide via the mitochondrial-dependent pathway in human prostate cancer cells. Cancer Chemother. Pharmacol..

[40] Misra S., Niyogi S. (2009). Selenite causes cytotoxicity in rainbow trout (*Oncorhynchus mykiss*) hepatocytes by inducing oxidative stress. Toxicol. In Vitro.

[41] Alanne M., Kristiansson K., Auro K., Silander K., Kuulasmaa K., Peltonen L., Salomaa V., Perola M. (2007). Variation in the selenoprotein s gene locus is associated with coronary heart disease and ischemic stroke in two independent finnish cohorts. Hum. Genet..

[42] Bailey R.L., Gahche J.J., Lentino C.V., Dwyer J.T., Engel J.S., Thomas P.R., Betz J.M., Sempos C.T., Picciano M.F. (2011). Dietary supplement use in the united states, 2003–2006. J. Nutr..

[43] Bermano G., Nicol F., Dyer J.A., Sunde R.A., Beckett G.J., Arthur J.R., Hesketh J.E. (1995). Tissue-specific regulation of selenoenzyme gene expression during selenium deficiency in rats. Biochem. J..

[44] Labunskyy V.M., Lee B.C., Handy D.E., Loscalzo J., Hatfield D.L., Gladyshev V.N. (2011). Both maximal expression of selenoproteins and selenoprotein deficiency can promote development of type 2 diabetes-like phenotype in mice. Antioxid. Redox Signal..

[45] Carlson B.A., Moustafa M.E., Sengupta A., Schweizer U., Shrimali R., Rao M., Zhong N., Wang S., Feigenbaum L., Lee B.J. (2007). Selective restoration of the selenoprotein population in a mouse hepatocyte selenoproteinless background with different mutant selenocysteine tRNAs lacking UM34. J. Biol. Chem..

[46] Moustafa M.E., Carlson B.A., El-Saadani M.A., Kryukov G.V., Sun Q.A., Harney J.W., Hill K.E., Combs G.F., Feigenbaum L., Mansur D.B. (2001). Selective inhibition of selenocysteine tRNA maturation and selenoprotein synthesis in transgenic mice expressing isopentenyladenosine-deficient selenocysteine tRNA. Mol. Cell. Biol..

[47] Leopold J.A., Loscalzo J. (2009). Oxidative risk for atherothrombotic cardiovascular disease. Free Radic. Biol. Med..

[48] De Lorgeril M., Salen P. (2006). Selenium and antioxidant defenses as major mediators in the development of chronic heart failure. Heart Fail. Rev..

[49] Toufektsian M.C., Boucher F., Pucheu S., Tanguy S., Ribuot C., Sanou D., Tresallet N., de Leiris J. (2000). Effects of selenium deficiency on the response of cardiac tissue to ischemia and reperfusion. Toxicology.

[50] Rakotovao A., Tanguy S., Toufektsian M.C., Berthonneche C., Ducros V., Tosaki A., de Leiris J., Boucher F. (2005). Selenium status as determinant of connexin-43 dephosphorylation in *ex vivo* ischemic/reperfused rat myocardium. J. Trace Elem. Med. Biol..

[51] Xia Y.M., Hill K.E., Burk R.F. (1985). Effect of selenium deficiency on hydroperoxide-induced glutathione release from the isolated perfused rat heart. J. Nutr..

[52] Ji L.L., Stratman F.W., Lardy H.A. (1992). Antioxidant enzyme response to selenium deficiency in rat myocardium. J. Am. Coll. Nutr..

[53] Doroshow J.H., Locker G.Y., Myers C.E. (1980). Enzymatic defenses of the mouse heart against reactive oxygen metabolites: Alterations produced by doxorubicin. J. Clin. Investig..

[54] Dimitrov N.V., Hay M.B., Siew S., Hudler D.A., Charamella L.J., Ullrey D.E. (1987). Abrogation of adriamycin-induced cardiotoxicity by selenium in rabbits. Am. J. Pathol..

[55] Nakano E., Takeshige K., Toshima Y., Tokunaga K., Minakami S. (1989). Oxidative damage in selenium deficient hearts on perfusion with adriamycin: Protective role of glutathione peroxidase system. Cardiovasc. Res..

[56] Boucher F., Coudray C., Tirard V., Barandier C., Tresallet N., Favier A., de Leiris J. (1995). Oral selenium supplementation in rats reduces cardiac toxicity of adriamycin during ischemia and reperfusion. Nutrition.

[57] Dursun N., Taskin E., Yerer Aycan M.B., Sahin L. (2011). Selenium-mediated cardioprotection against adriamycin-induced mitochondrial damage. Drug Chem. Toxicol..

[58] Van Vleet J.F., Ferrans V.J., Weirich W.E. (1980). Cardiac disease induced by chronic adriamycin administration in dogs and an evaluation of vitamin E and selenium as cardioprotectants. Am. J. Pathol..

[59] Hermansen K., Wassermann K. (1986). The effect of vitamin E and selenium on doxorubicin (adriamycin) induced delayed toxicity in mice. Acta Pharmacol. Toxicol..

[60] Lymbury R.S., Marino M.J., Perkins A.V. (2010). Effect of dietary selenium on the progression of heart failure in the ageing spontaneously hypertensive rat. Mol. Nutr. Food Res..

[61] Bhattacharya A., Turowski S.G., San Martin I.D., Rajput A., Rustum Y.M., Hoffman R.M., Seshadri M. (2011). Magnetic resonance and fluorescence-protein imaging of the anti-angiogenic and anti-tumor efficacy of selenium in an orthotopic model of human colon cancer. Anticancer Res..

[62] Liu J.G., Zhao H.J., Liu Y.J., Wang X.L. (2010). Effect of selenium-enriched malt on VEGF and several relevant angiogenic cytokines in diethylnitrosamine-induced hepatocarcinoma rats. J. Trace Elem. Med. Biol..

[63] Wang Z., Hu H., Li G., Lee H.J., Jiang C., Kim S.H., Lu J. (2008). Methylseleninic acid inhibits microvascular endothelial G1 cell cycle progression and decreases tumor microvessel density. Int. J. Cancer.

[64] Raines A.M., Sunde R.A. (2011). Selenium toxicity but not deficient or super-nutritional selenium status vastly alters the transcriptome in rodents. BMC Genomics.

[65] Bajpai S., Mishra M., Kumar H., Tripathi K., Singh S.K., Pandey H.P., Singh R.K. (2011). Effect of selenium on connexin expression, angiogenesis, and antioxidant status in diabetic wound healing. Biol. Trace Elem. Res..

[66] Wang Z., Jiang C., Ganther H., Lu J. (2001). Antimitogenic and proapoptotic activities of methylseleninic acid in vascular endothelial cells and associated effects on PI3K-AKT, ERK, JNK and p38 MAPK signaling. Cancer Res..

[67] Tang R., Liu H., Wang T., Huang K. (2005). Mechanisms of selenium inhibition of cell apoptosis induced by oxysterols in rat vascular smooth muscle cells. Arch. Biochem. Biophys..

[68] Huang Z., Rose A.H., Hoffmann P.R. (2012). The role of selenium in inflammation and immunity: From molecular mechanisms to therapeutic opportunities. Antioxid. Redox Signal..

[69] Rivera M.T., de Souza A.P., Moreno A.H., Xavier S.S., Gomes J.A., Rocha M.O., Correa-Oliveira R., Neve J., Vanderpas J., Araujo-Jorge T.C. (2002). Progressive Chagas cardiomyopathy is associated with low selenium levels. Am. J. Trop. Med. Hyg..

[70] Davis C.D., Brooks L., Calisi C., Bennett B.J., McElroy D.M. (1998). Beneficial effect of selenium supplementation during murine infection with trypanosoma cruzi. J. Parasitol..

[71] De Souza A.P., de Oliveira G.M., Vanderpas J., de Castro S.L., Rivera M.T., Araujo-Jorge T.C. (2003). Selenium supplementation at low doses contributes to the decrease in heart damage in experimental trypanosoma cruzi infection. Parasitol. Res..

[72] Stoedter M., Renko K., Hog A., Schomburg L. (2010). Selenium controls the sex-specific immune response and selenoprotein expression during the acute-phase response in mice. Biochem. J..

[73] Kucharz E.J., Olczyk K. (1993). Influence of chronic intoxication with selenium on collagen and elastin content in tissues of rat. Toxicol. Lett..

[74] Ding M., Potter J.J., Liu X., Torbenson M.S., Mezey E. (2010). Selenium supplementation decreases hepatic fibrosis in mice after chronic carbon tetrachloride administration. Biol. Trace Elem. Res..

[75] Chen J., Chu Y., Cao J., Wang W., Liu J., Wang J. (2011). Effects of T-2 toxin and selenium on chondrocyte expression of matrix metalloproteinases (MMP-1, MMP-13), α2-macroglobulin (α2M) and timps. Toxicol. In Vitro.

[76] Yoon S.O., Kim M.M., Chung A.S. (2001). Inhibitory effect of selenite on invasion of HT1080 tumor cells. J. Biol. Chem..

[77] Carlson B.A., Yoo M.H., Sano Y., Sengupta A., Kim J.Y., Irons R., Gladyshev V.N., Hatfield D.L., Park J.M. (2009). Selenoproteins regulate macrophage invasiveness and extracellular matrix-related gene expression. BMC Immunol..

[78] Handy D.E., Castro R., Loscalzo J. (2011). Epigenetic modifications: Basic mechanisms and role in cardiovascular disease. Circulation.

[79] Cox R., Goorha S. (1986). A study of the mechanism of selenite-induced hypomethylated DNA and differentiation of friend erythroleukemic cells. Carcinogenesis.

[80] Xiang N., Zhao R., Song G., Zhong W. (2008). Selenite reactivates silenced genes by modifying DNA methylation and histones in prostate cancer cells. Carcinogenesis.

[81] Zeng H., Yan L., Cheng W.H., Uthus E.O. (2011). Dietary selenomethionine increases exon-specific DNA methylation of the p53 gene in rat liver and colon mucosa. J. Nutr..

[82] Davis C.D., Uthus E.O., Finley J.W. (2000). Dietary selenium and arsenic affect DNA methylation *in vitro* in Caco-2 cells and *in vivo* in rat liver and colon. J. Nutr..

[83] Davis C.D., Uthus E.O. (2002). Dietary selenite and azadeoxycytidine treatments affect dimethylhydrazine-induced aberrant crypt formation in rat colon and DNA methylation in HT-29 cells. J. Nutr..

[84] Novoselov S.V., Calvisi D.F., Labunskyy V.M., Factor V.M., Carlson B.A., Fomenko D.E., Moustafa M.E., Hatfield D.L., Gladyshev V.N. (2005). Selenoprotein deficiency and high levels of selenium compounds can effectively inhibit hepatocarcinogenesis in transgenic mice. Oncogene.

[85] Joseph J., Joseph L. (2003). Hyperhomocysteinemia and cardiovascular disease: New mechanisms beyond atherosclerosis. Metab. Syndr. Relat. Disord..

[86] Hill K.E., Burk R.F. (1982). Effect of selenium deficiency and vitamin E deficiency on glutathione metabolism in isolated rat hepatocytes. J. Biol. Chem..

[87] Hill K.E., Burk R.F., Lane J.M. (1987). Effect of selenium depletion and repletion on plasma glutathione and glutathione-dependent enzymes in the rat. J. Nutr..

[88] Hassoun B.S., Palmer I.S., Dwivedi C. (1995). Selenium detoxification by methylation. Res. Commun. Mol. Pathol. Pharmacol..

[89] Rajasekaran N.S., Connell P., Christians E.S., Yan L.J., Taylor R.P., Orosz A., Zhang X.Q., Stevenson T.J., Peshock R.M., Leopold J.A. (2007). Human αB-crystallin mutation causes oxido-reductive stress and protein aggregation cardiomyopathy in mice. Cell.

[90] McClung J.P., Roneker C.A., Mu W., Lisk D.J., Langlais P., Liu F., Lei X.G. (2004). Development of insulin resistance and obesity in mice overexpressing cellular glutathione peroxidase. Proc. Natl. Acad. Sci. USA.

[91] Wang X.D., Vatamaniuk M.Z., Wang S.K., Roneker C.A., Simmons R.A., Lei X.G. (2008). Molecular mechanisms for hyperinsulinaemia induced by overproduction of selenium-dependent glutathione peroxidase-1 in mice. Diabetologia.

[92] Yan X., Pepper M.P., Vatamaniuk M.Z., Roneker C.A., Li L., Lei X.G. (2012). Dietary selenium deficiency partially rescues type 2 diabetes-like phenotypes of glutathione peroxidase-1-overexpressing male mice. J. Nutr..

[93] Cockerham C.C. (1954). An extension of the concept of partitioning hereditary variance for analysis of covariances among relatives when epistasis is present. Genetics.

[94] Loscalzo J., Barabasi A.L. (2011). Systems biology and the future of medicine. Wiley Interdiscip. Rev. Syst. Biol. Med..

[95] Van Ommen B., Fairweather-Tait S., Freidig A., Kardinaal A., Scalbert A., Wopereis S. (2008). A network biology model of micronutrient related health. Br. J. Nutr..

[96] Van Ommen B., El-Sohemy A., Hesketh J., Kaput J., Fenech M., Evelo C.T., McArdle H.J., Bouwman J., Lietz G., Mathers J.C. (2010). The micronutrient genomics project: A community-driven knowledge base for micronutrient research. Genes Nutr..

[97] Wikipathway Web site.

